# Adolescent sexual and reproductive health and rights for ethnic minority girls in Vietnam

**DOI:** 10.1093/heapro/daad182

**Published:** 2024-01-17

**Authors:** Lia Burns, Hannah Pitt, Thuy Luu Ngoc

**Affiliations:** Institute for Health Transformation, Faculty of Health, Deakin University, 301 Burwood Highway, Burwood, Victoria, 3125, Australia; Institute for Health Transformation, Faculty of Health, Deakin University, 301 Burwood Highway, Burwood, Victoria, 3125, Australia; Social Policy Research Centre (SPRC), Arts, Design & Architecture, University of New South Wales, High Street, Kensington, New South Wales, 2052, Australia

**Keywords:** adolescent sexual and reproductive health, Vietnam, ethnic minority girls, social determinants of health, social norms

## Abstract

There has been significant progress in improved adolescent sexual and reproductive health and rights (ASRHR) for girls across low- to- middle-income countries (LMICs). However, progress has been uneven, and disparities persistent across and within countries. For example, Vietnam is one of only nine countries to have achieved the 2015 maternal mortality rate (MMR) target of the Millennium Development Goals (MDGs) as a nation, but for some sub-populations, progress has been much slower, and MMR is more than twice that the national average. Ensuring equity is a current focus of the Sustainable Development Goals (SDGs) agenda that seeks to Leave No One Behind. This paper explores some of these inequities and potential drivers for ethnic minority adolescent girls in Vietnam, with a specific focus on the Tay community, the largest ethnic minority group in Vietnam. This paper highlights the challenges to progress, including areas where there is still limited evidence about the range of socio-cultural factors that may determine sexual and reproductive health outcomes for Tay adolescent girls. In the era of the SDGs, Vietnam’s national policy platforms and current aid architecture provide a solid basis on which to build research, policy and practice investments that improve the health of adolescent ethnic minority girls in Vietnam.

**Contribution to Health Promotion**

- This paper considers socio-cultural determinants that contribute to health disparities and inhibit effective responses to the sexual and reproductive health needs of adolescent ethnic minority girls in Vietnam.
- A range of persisting socio-cultural and structural factors may compromise the sexual and reproductive health of Tay adolescent girls.
- Actions to address this challenge must consider the rapidly changing socio-economic environment in Vietnam and the experiences of ethnic minority groups.
- Health promotion initiatives should prioritize the lived experience of adolescent girls, ensuring that local knowledge is combined with international best practices to develop culturally aligned adolescent sexual and reproductive health responses.

## ADOLESCENT SEXUAL AND REPRODUCTIVE HEALTH AND RIGHTS: A GLOBAL PUBLIC HEALTH ISSUE

As a basic human right, the concept of adolescent sexual and reproductive health and rights (ASRHR) is articulated in the United Nations Sustainable Development Goal (SDG) 3.7, which aims for universal access to sexual and reproductive health and rights for all by 2030 (United Nations, n.d.). The Lancet Commission on Adolescent Health and Wellbeing (Patton *et al*., 2016) states that adolescence is the period of life for girls and boys between 10 and 24 years, with 1.3 billion adolescents making up roughly 16% of the world’s population (UNICEF 2022). The age range of 10–24 years old is used for this developmental phase of the adolescent girl (Sawyer *et al*., 2018).

The United National Population Development Fund (UNDP, 2022:16) defines sexual and reproductive health (SRH) as a state of ‘*complete physical, mental, and social well-being in all matters relating to the reproductive system’.* For adolescents, this definition implies access to accurate information, education, counselling, and safe, effective, affordable and acceptable health services in recognition of the high risks of early pregnancy, unsafe abortion, sexually transmitted diseases, reproductive and sexual coercion (Wood *et al*., 2023) and associated mental health issues (UNDP, 2022; WHO, n.d.). This paper uses the term reproductive health to encompass this broad definition for adolescents.

Since the 1994 International Conference on Population and Development (ICPD), increased investments have seen global gains in adolescent reproductive health. Improvements relating to adolescents initiating sexual activity later and being more likely to use a condom during sex (WHO, 2020) contributed to the decline of global adolescent fertility rates from 64.5 births per 1000 girls (15–19 years) in 2000 to 41.3 births per 1000 girls in 2023 (WHO, 2023b). However, significant challenges remain for girls, with approximately 14% of girls globally giving birth before they are 18 years old (UNICEF, 2022). Following the end of the Millennium Development Goals 2000–2015 (MDGs) (WHO, 2018), the SDGs 2015 to 2030 (United Nations, 2020) highlighted the need for a greater focus on adolescents as an overall demographic, particularly girls (Asha *et al*., 2021). This included calls for a significant increase in global investment to improve outcomes for this group (Chandra-Mouli *et al*., 2019) with attention to:

(i) legal frameworks that fail to uphold the right to health for all adolescents.
(ii) health services that are not responsive to the needs of adolescents, girls in particular.
(iii) harmful socio-cultural factors relating to the sexuality of adolescent girls.

Therefore, reductions across important reproductive health indicators have occurred (e.g. unintended pregnancies) (United Nations, 2020), but persistent socio-cultural and structural issues continue to challenge effective delivery of policy and services. For example, cultural norms perpetuating the acceptance of early marriage of girls and maintaining taboos relating to pre-marital sex continue to inhibit uptake of low-quality and poorly resourced reproductive health services (Chi, 2021; Dawkins *et al.*, 2021; WHO, 2021; Akinwale *et al*., 2022). The WHO Global Strategy for Women’s, Children’s and Adolescent Health (2016–2030) guides country-level policy and planning, and highlights the centrality and importance of a focus on adolescents, who have, historically, been neglected in global and national policy responses (World Health Organisation, 2015).

## THE REPRODUCTIVE HEALTH OF GIRLS IN LMICs

In this paper, we use the term low- to- middle-income countries (LMICs) to illustrate how a level of national economic development co-existing with diverse socio-cultural values can exhibit a range of socio-economic inequalities for some groups, for example, self-determination and access to social services for girls (Ngoc Do *et al*., 2020; Le and Yu, 2021; Patel *et al*., 2022). This plays out differently across countries and sub-populations where rapid socio-economic development, the uptake of technology and pressures of cultural globalization are interplaying with cultural traditions (Watson, 2023).

Over the last 10 years, global policy platforms addressing the reproductive health of adolescents have seen improvements in some areas, such as a decline in the adolescent fertility rate (WHO, 2023a), however, progress is slow for some populations, health issues and geographic regions (Chandra-Mouli *et al*., 2019). For example, the SDG’s (United Nations, 2020) and the WHO Global Strategy (World Health Organisation, 2015) specifically champion the needs of adolescent girls in LMICs who still experience the highest global prevalence of unintended pregnancies amongst 10–19 years old in the context of complex socio-cultural contexts. These girls have unmet reproductive health needs including prevention and management of early and unintended pregnancy, complications relating to abortion, menstrual hygiene management and sexual violence (UNFPA. 2021). In 2019, there were 21 million pregnancies for girls between 15 and 19 years old, of which 50% were unintended (WHO, 2023). Negative effects of early or unintended pregnancy can include compromised health during pregnancy and childbirth, reduced education and socio-economic opportunities, poor psychological health and diminished life potential for mother and child (Aggarwal *et al*., 2022). A 2022 meta-synthesis of 21 qualitative studies across LMICs concluded that culture and religion can have a distinct influence and control over a range of reproductive health issues affecting girls, including their fertility, where pregnancy was often associated with negative emotions and/or feeling unready for motherhood (Crooks *et al*., 2022).

Across the Asia Pacific region, the implications of negative reproductive health outcomes are greater for adolescent girls than boys (Kennedy *et al*., 2020), and affect not only the future adult life of girls but also their children (Patton *et al*., 2016). Just under half a billion adolescent girls live in this region (Kennedy *et al*., 2020) and research shows that intergenerational social norms play a key role in reproductive health outcomes for girls (Chandra-Mouli *et al.*, 2019). Chandra-Mouli *et al.* (2019) argue that family and community strongly influence how social and gender norms empower or disempower girls in their health seeking behaviours. Furthermore, social and family sanctions (Bicchieri and Mercier, 2014) can influence the balance of power between males and females, perpetuating gender norms that are contextually realized depending on age, reproductive life stage, life course and partner status (Gillespie *et al.*, 2022). For example, studies showed that normative decision making related to early marriage often occurs between senior family members for social or economic reasons, with no or limited involvement of the girl (Santhya and Jejeebhoy, 2015; Udgiri 2017; Girls Not Brides n.d). While social norms may impact the interaction between parents and their children in addressing sensitive topics (Kaljee *et al*., 2011), recent studies have shown significant possibilities for mothers to act within their own socio-cultural contexts to improve health outcomes for their daughters (Asadullah and Wahhaj, 2019; Van Bavel, 2020).

## THE REPRODUCTIVE HEALTH OF GIRLS IN VIETNAM

The *‘Doi Moi’* economic reforms of the 1980s saw a transition in the concept of adolescence in Vietnam (population 98million), from a stage of political maturity to a discrete and important time of the human life cycle (Nguyen, 2015). This evolving concept, coupled with ongoing social and economic development, contests new values against more traditional norms related to gender (Bui, 2020) and the sexuality of girls (Hoang *et al*., 2018; UNICEF & UNFPA n.d). Consequently, ongoing issues relating to the reproductive health of approximately 7 million adolescent girls or 7% of the nation’s total population, include a range of persistent challenges associated with the social determinants of health (UNFPA, 2023).

Vietnam is characterized as a collective culture with strong patriarchal values across 54 ethnic groups with the majority Kinh (Viet) consisting of 85% of the national population (Government of Vietnam, 2017). The remaining 15% populates 53 ethnic minority groups who experience vulnerabilities due to complex and inter-related factors of ethnicity, geographic isolation, cultural and linguistic differences and multi sectoral poverty (Government of Vietnam, 2017). Vietnam is one of only nine countries to have achieved the 2015 maternal mortality rate (MMR) target of the MDG’s 2000–2015 (UNFPA, 2023), however, socio-cultural practices negatively affecting the health of women and girls are still common. UN Women report that girls and women across all populations in Vietnam are the most socio-economically disadvantaged due to weak enforcement of laws and deep rooted social and gender norms limiting agency and opportunity (UN Women, n.d). National level reporting (General Statistics Office and UNICEF, 2021) and study findings over the last 25 years all highlight risky sexual behaviours, unintended pregnancy and repeat abortion, as the major ongoing reproductive health issues for Vietnamese girls (Gorbach *et al.,* 1998; Kaljee *et al.,* 2007; Teerawichitchainan and Amin, 2010; Ngoc *et al*., 2020). A 2015 literature review (Vinh, 2015) also concluded that while factors occurring across the full socio-ecological framework were inter-related, interpersonal social norms stigmatizing pre-marital sex were of significant consequence. These findings illustrate a society in transition where adolescent girls are increasingly practising pre-marital sex within the context of low interpersonal and broader structural support to ensure positive reproductive health outcomes (General Statistics Office and UNICEF, 2021).

Vietnam is unique in the Southeast Asian region due to the rapid and stable socio-economic reform that has raised the majority of its population out of poverty in one generation (World Bank; Ministry of Planning and Investment of Vietnam, 2016). The Government of Vietnam (GoV), United Nations and civil society partners are now addressing remaining barriers that are widening disparities for some child and adolescent populations driven by ethnicity, gender, place of origin and disability (UNICEF, n.d). The GoV has shown strong political will to successfully address complex health challenges, for example, the reduction in maternal mortality through the successful Safe Motherhood National Plan (Ha *et al*., 2015). The characteristics of the agenda setting in this scenario illustrates the potential for further public health gains whereby (i) the problem is recognized by policy makers with evidence from high level research; (ii) GoV policy champions are supported by development partners and (iii) domestic and international events promote the agenda (Yang and Qian, 2016). With this state of readiness, further research on child and adolescent populations and trial interventions has the potential for high return on investment and can provide exemplars to other countries with similar socio-economic trajectories and characteristics.

A major challenge in improving policy responses relating to the reproductive health of adolescent girls in Vietnam is the current lack of a comprehensive strategy or plan addressing the health of adolescents as a specific population with discrete needs (Chi, 2021). A cohesive national strategy, locally adapted to global best practice, could strengthen the current response where weak and sporadic, uncoordinated, under-resourced and poorly implemented services are common (WHO, 2019). For example, a 2021 study on ASRHR policies and programs in Vietnam found that comprehensive sexuality education is not mandatory in schools, no specific guidelines or plans to address gender inequities across services are in place, health worker capacity is under-resourced and guidance on service provision for disadvantaged groups was insufficient and unspecified (Chi, 2021). Chi (2021) recommended that future policy reform should focus on interventions for disadvantaged groups including ethnic minorities but did not address how.

One of the most significant issues facing improved reproductive health responses for girls in Vietnam is that most evidence for this population is only available for the majority Kinh population. Barriers to health information collection in ethnic minority areas have included geographical isolation, linguistic and cultural barriers and lack of political and financial commitment (Malqvist *et al*., 2013). Malqvist (2013) also reported a lack of analysis on ethnicity from major national surveys and census. Only recently has data on ethnic minority girls begun to consistently emerge in national level data sets (General Statistics Office and UNICEF, 2021). Common findings across national and community level data all relate to health inequities between the majority and minority populations but detail on the reproductive health of ethnic minority girls is still relatively scarce(Chi, 2021; Le and Yu, 2021).

## THE REPRODUCTIVE HEALTH OF ETHNIC MINORITY GIRLS IN VIETNAM

In Vietnam, progress made nationally in sexual and reproductive health is offset by ongoing disparities in ethnic minority populations, where, for example, the MMR is 2–3 times higher for girls in these groups (UNFPA, 2023). In Vietnam, ethnic minority communities are generally poorer, live in remote and/or mountainous areas, and have limited access to health and education services (Malqvist *et al.*, 2013) affecting girls across important indicators (General Statistics Office and UNICEF, 2021). Provision and uptake of reproductive health services is ineffective due to low national budget allocation, limited transportation options, cultural and linguistic differences between health workers and clients, poorly resourced health facilities and service delivery capacity (Malqvist *et al*., 2013; UNFPA, 2022). Malqvist (2013) concluded that known barriers were compounded for girls where the socio-cultural determinants of harmful social norms and patriarchal structures affected health care choices and outcomes. A more recent report also reiterated that health service policies and programs do not respond to contextual challenges that perpetuate health disparities between ethnic minority girls and girls from the majority Kinh population (UN Women, 2021).

For ethnic minority girls in Vietnam, intersectional complexities related to ethnicity, gender and age contribute to health inequities and poor reproductive health (Thi *et al*., 2023). For example, Lee and Yu (2021) found a range of challenges faced by unmarried 19- to 22-year-old girls from nine ethnic groups in the Northern Uplands of Vietnam, including negative experiences related to accessing SRH services, stigma and discrimination, and inadequate understanding of contraception and sexually transmitted diseases. National level health policies, strategies and academic literature recognize that health inequities for ethnic minority groups are influenced by cultural traditions and social norms in these communities. However, health service programming and investment in Vietnam are still challenged by limited understanding of how these phenomena contribute to poor reproductive health outcomes for ethnic minority girls (Malqvist *et al*., 2013; Le and Yu, 2021; Nowshin *et al*., 2022).

Fortunately, there are opportunities for action within current policy platforms. The GoV’s Leave No One Behind Sustainable Development Goals Action Plan (Government of Vietnam, 2017) prioritizes all ethnic minority groups in recognition of the persistent multi-dimensional poverty rates of these populations (Government of Vietnam, 2021). International organizations and local civil society also prioritize ethnic minority girls for funding and programming in the context that ethnic minority populations maintain the lowest socio-economic status in Vietnam, despite rapid economic growth across the country in recent decades (World Bank Group, 2019).

One way of addressing this knowledge gap may be to work with the Tay community—the largest ethnic minority group in Vietnam (Hien *et al*., 2021). The Tay live predominantly in the northern mountainous regions of Vietnam, across five sub-groups in six provinces, Bac Kan, Cao Bang, Hoa Binh, Lang Son, Quang Ninh, Ha Giang (General Statistics Office, 2019). While evidence about the health and wellbeing of the Tay is limited, population level reports over the last 5 years have shown that there are considerable inequities in access to health services (WHO, 2019; General Statistics Office and UNICEF, 2021), with poor health outcomes similar across ethnic groups (Malqvist *et al.*, 2013; Hoang *et al*., 2018; Le and Yu, 2021). In relation to the reproductive health of adolescent girls, national level data specifically mentioned high rates of induced abortion in Tay-headed households (General Statistics Office and UNICEF, 2021), with studies reporting that Tay girls’ experience stigma related to sexuality and have a limited understanding of how to manage their fertility (Lee and Yu, 2021), and married Tay women report that they do not use contraception because their husbands do not agree (Luu *et al.*, 2023).

Several studies report on consanguineous marriage in Tay communities (Trang and Nguyen, 2015; Minh, 2019) and the risks of thalassaemia (Anh *et al*., 2019), with some estimating that over three quarters of Tay marriages are consanguineous (Trang and Nguyen, 2015). Given the link between early and consanguineous marriage and an increased risk of autosomal recessive disorders and congenital anomalies (Minh, 2019), there is a particular need for intervention with the Tay community. Research from 2022 shows that over 13.8% of the population of Vietnam carry thalassaemia genes (Bach *et al*., 2022). Anh et al. (2019) cites studies that report a relatively lower prevalence among the Kinh population compared to that of ethnic minority groups with 25% of Tay children likely to inherit and 50% will carry, a genetic disposition for the haemolytic disease. In Tay communities, the prevalence of thalassaemia major, a congenital blood disorder causing chronic severe anaemia in children is significant, and morbidity and mortality rates are high (Anh *et al*., 2019; Bach *et al*., 2022; Riquier, 2022). Health programs providing information and education about this condition are virtually non-existent in these communities (Bach *et al*., 2022). In addition, pre-marital or pre-conception screening is not easily accessible, and centrally located treatment options are prohibitively expensive and difficult to access (Anh *et al.*, 2019; Bach *et al*., 2022). The implications for Tay girls are multiple and this scenario shows how in collective cultures where consanguineous, early and/or arranged marriages are accepted and girls have limited reproductive autonomy, access to health resources or decision making, the likelihood of offspring with an inherited disease is high.

Furthermore, for adolescent ethnic minority populations, multiple external pressures on their traditional way of life from rural to urban living, socio-economic development and increasing access to the internet is influencing socio-cultural norms and values. For example, reporting from Vietnam (UN Women, 2020) and studies from Ethiopia and India (McDougal *et al.,* 2018) have found that the agency of adolescents and young people in decision making is shifting in relation to marriage and sexual practices. This indicates a shift in what we have traditionally understood about the self-determination of girls in collective cultures like Vietnam (UN Women, 2020).

## AREAS FOR ACTION IN RESEARCH, POLICY AND PRACTICE

The current reproductive health challenges facing adolescent girls in Vietnam, show how girls can experience greater health inequities due to socio-cultural barriers that may not be well understood (Malqvist *et al*., 2013; Binder-Finnema *et al*., 2015; UNFPA, 2022). For example, why are there socio-cultural restrictions to girls accessing reproductive health care? Are there perceived socio-cultural benefits? The term ‘last mile’ has been applied to adolescent populations in LMICs, including ethnic minorities, to highlight there is still action needed to address the gaps in the evidence base to reduce health inequities for these groups (Nowshin *et al.*, 2022). This sentiment also underpins the SDG ‘Leave No One Behind’ partnership in Vietnam (MSD Vietnam, 2021), which seeks to understand the specific needs of ethnic minority populations for targeted investments and interventions.

The range of research, policy and practice needs for adolescent reproductive health articulated at the global level are also relevant for Vietnam. For example, updating what we know about how ongoing socio-economic change affects social and gender norms (OECD, 2021), and the need to contextualize Eurocentric health promotion strategies (Wazir, 2022; Newman, 2023). The research agenda would benefit from increased demand by the GoV for contextualized global best practice methods in public health, as the basis for a comprehensive national ASRHR policy. Here we suggest two areas for action. First is collaboration on formative work between research institutions/universities and civil society organizations which could provide baseline data on socio-cultural factors that underpin sector/program strategy, theory of change, program, project design, and monitoring and evaluation. Second, is the role of national, United Nations and bilateral funding agencies in investing in program development with sufficient lead-time planning, flexible funding and simpler administration necessary for these collaborations to be meaningful and effective.

An important next step for GoV’s policy platform is to prioritize how policy and programming is developed, and on what evidence basis (Newman, 2023). Through new formative work, the current disparate policies addressing adolescent reproductive health could be combined into one policy including feasible and contextualized socio-cultural responses across prevention, promotion and service delivery components of the health system. Collaborators could, for example, undertake formative work to inform prevention of early marriage strategies, exploring the myriad of reasons for early marriage across different communities in Vietnam and address the socio-economic factors perpetuating early marriage, not just the harmful outcomes.

Practice would benefit from partnerships between international and local development organizations that specifically promotes and supports local leadership to build locally led responses. This should include gender-balanced community representation and youth-led problem identification and creation of solutions. To steward this process, cross pollination of skills between researchers and practitioners will require institutional funding, support and co-operation. A pilot investment is due in joint skills development on formative work to better understand socio-cultural norms affecting the health of ethnic minority girls. Using a locally led partnership between public health researchers and public health practitioners, with implementation research techniques (Theobald *et al.*, 2018) along with flexible timelines and budgets may also provide an example model for future approaches globally.

The confluence of the current knowledge base on ASRHR and the decolonization agenda, provides the perfect foundation to improve public health responses for all adolescent girls. For ethnic minority girls in Vietnam, researchers and practitioners can find ways to better engage with this group. Expanding our understanding and response to the socio-cultural determinants of girl’s health is possible. The right research, policy and practice choices can inherently contribute to reducing health inequities and improve health outcomes for disadvantaged populations including adolescent ethnic minority girls in Vietnam.
